# Photostabilization of Poly(vinyl chloride) by Organotin(IV) Compounds against Photodegradation

**DOI:** 10.3390/molecules24193557

**Published:** 2019-10-01

**Authors:** Angham G. Hadi, Khudheyer Jawad, Gamal A. El-Hiti, Mohammad Hayal Alotaibi, Ahmed A. Ahmed, Dina S. Ahmed, Emad Yousif

**Affiliations:** 1 Department of Chemistry, College of Science, Babylon University, Babil 51002, Iraq; analhusainy@gmail.com (A.G.H.); khudheyer1965@gmail.com (K.J.); 2 Cornea Research Chair, Department of Optometry, College of Applied Medical Sciences, King Saud University, P.O. Box 10219, Riyadh 11433, Saudi Arabia; 3 National Center for Petrochemicals Technology, King Abdulaziz City for Science and Technology, P.O. Box 6086, Riyadh 11442, Saudi Arabia; 4 Polymer Research Unit, College of Science, Al-Mustansiriyah University, Baghdad 10052, Iraq; ahmedahmedalazawi@gmail.com; 5 Department of Medical Instrumentation Engineering, Al-Mansour University College, Baghdad 64021, Iraq; dinasaadi86@gmail.com; 6 Department of Chemistry, College of Science, Al-Nahrain University, Baghdad 64021, Iraq

**Keywords:** telmisartan organotin(IV) compounds, poly(vinyl chloride), ultraviolet irradiation, photodegradation, polymer weight loss, surface morphology

## Abstract

Poly(vinyl chloride) (PVC), a polymer widely used in common household and industrial materials, undergoes photodegradation upon ultraviolet irradiation, leading to undesirable physicochemical properties and a reduced lifetime. In this study, four telmisartan organotin(IV) compounds were tested as photostabilizers against photodegradation. PVC films (40-µm thickness) containing these compounds (0.5 wt%) were irradiated with ultraviolet light at room temperature for up to 300 h. Changes in various polymeric parameters, including the growth of hydroxyl, carbonyl, and alkene functional groups, weight loss, reduction in molecular weight, and appearance of surface irregularities, were investigated to test the efficiency of the photostabilizers. The changes were more noticeable in the blank PVC film than in the films containing the telmisartan organotin(IV) compounds. These results reflect that these compounds effectively inhibit the photodegradation of PVC, possibly by acting as hydrogen chloride and radical scavengers, peroxide decomposers, and primary photostabilizers. The synthesized organotin(IV) complexes could be used as PVC additives to enhance photostability.

## 1. Introduction

Poly(vinyl chloride) (PVC) is a common and widely used plastic produced by the polymerization of vinyl chloride [1]. There are two main types of PVC, namely, rigid and flexible PVC [2]. Rigid PVC resists degradation better than flexible PVC and is more resistant to chemicals (e.g., acids and bases), water, fire, and weather [3]. For example, the addition of phthalate plasticizers to PVC during manufacturing produces soft PVC [4]. Moreover, the addition of wood flour fillers enhances its biodegradation. PVC is used in several industrial applications, such as the production of bottles, roofing sheets, floor coverings, pipes, cable insulation, packaging foils, and various medicinal products [5,6]. The most common advantages of PVC are its low production cost and good physical, mechanical, and chemical properties. However, PVC suffers severely from photodegradation at relatively high temperatures (above 100 °C), mainly owing to dehydrochlorination, which leads to the evolution of hydrogen chloride and the formation of polyene residues [7,8]. This process leads to undesirable changes in the physical (e.g., discoloration and brittleness) and chemical (e.g., cross-linking and branching) properties of PVC, and lessens its durability [9,10]. In landfill sites, PVC degradation can be enhanced by the addition of alkali in the presence of molecular oxygen at high temperature. To increase lifetime of PVC, photodegradation and photo-oxidation should be inhibited. Various heat stabilizers, ultraviolet (UV) absorbers, radical scavengers, and excited-state quenchers have been used to inhibit these processes [11,12,13,14,15,16,17,18,19,20,21,22].

Organotin(IV) compounds have unique chemical properties and structural diversity. They can act as catalysts and reducing reagents and interact with various biologically active compounds [23]. Tin can form a variety of stable complexes with many organic ligands containing electron-rich atoms (e.g., heteroatoms such as nitrogen, oxygen, sulfur, and phosphorous) [24]. The diversity and activity of these compounds are highly dependent on the number and type of substituents (e.g., aryl or alkyl moieties) attached to tin [24,25]. In addition, organotin(IV) compounds show antitumor, antibacterial, and antimalarial activities, and can be used as agricultural biocides [26,27,28]. Various organotin(IV) compounds containing aromatic moieties have been synthesized and used as efficient PVC photostabilizers against UV irradiation [29,30,31,32,33,34]. The photo-oxidation and photodegradation processes of PVC lead to the production of small fragments and the formation of low molecular weight residues, and undesirable change in color, physical, and chemical properties [35,36].

Recently, we reported the synthesis of novel organotin(IV) frameworks containing telmisartan and their use as carbon dioxide storage media [31]. These tin(IV) frameworks have a mesoporous structure, large surface area, large pore size, and high aromatic content [31]. In addition, such additives contain telmisartan as a ligand, which is a pharmaceutically active ingredient. Therefore, they are ideal for use as additives to protect PVC against irradiation. Herein, we report the successful use of telmisartan organotin(IV) frameworks as new effective PVC photostabilizers against photodegradation upon UV irradiation.

## 2. Results and Discussion

### 2.1. Organotin(IV) Compounds ***1**–**4***

**Figure 1:**
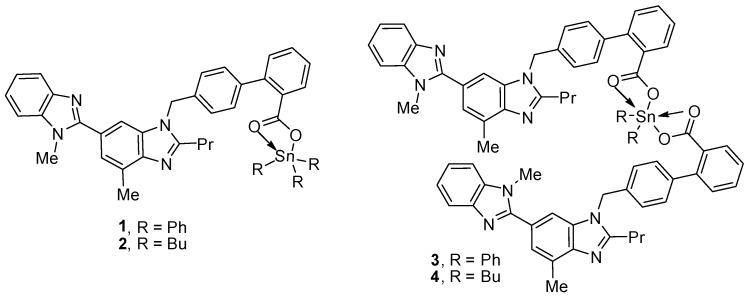
Organotin(IV) compounds **1**–**4**.

Triorganotin(IV) compounds **1** and **2** and diorganotin(IV) compounds **3** and **4** (Figure 1) were synthesized using known procedures [31]. The reaction of a 1:1 mixture of telmisartan and triphenyltin(IV) chloride or tributyltin(IV) chloride in boiling methanol for 8 h created **1** and **2**, respectively [31]. On the other hand, the reaction of a 2:1 mixture of telmisartan and diphenyltin(IV) chloride or dibutyltin(IV) chloride under similar reaction conditions created **3** or **4**, respectively. The spectral data of compounds **1**–**4** were consistent with the reported ones [31].

### 2.2. Fourier-Transform Infrared (FTIR) Spectroscopy of PVC

**Figure 2:**
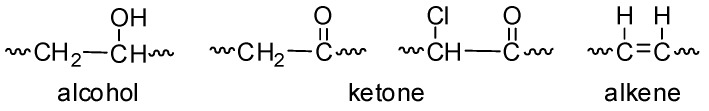
Alcohol, ketone, and alkene fragments formed by photo-oxidation of poly(vinyl chloride) (PVC).

**Figure 3:**
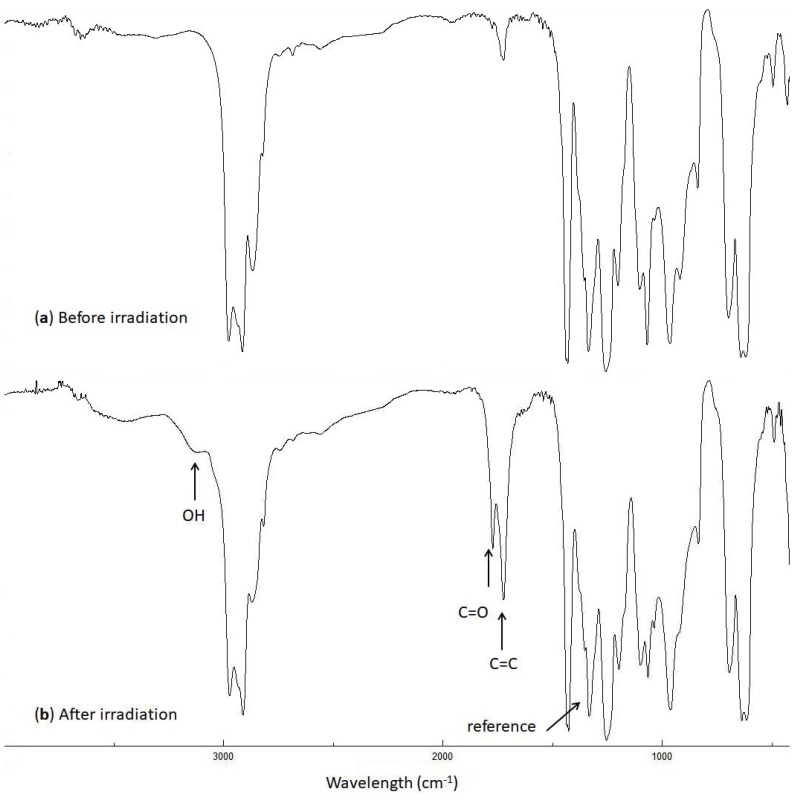
FTIR spectra of the blank PVC film (**a**) before and (**b**) after irradiation (300 h).

The photo-oxidation of PVC produces various fragments (Figure 2) that contain different functional groups (e.g., alcohol, ketone, and alkene) [16,35,36]. The intensities of these functional groups can be monitored by FTIR spectroscopy (400–4000 cm^−1^) of the irradiated PVC films. Therefore, the PVC films in the absence and presence of low concentrations (0.5 wt%) of compounds **1**–**4** were irradiated with UV light (λ = 365 nm and light intensity = 6.43 × 10^−9^ ein·dm^−3^·s^−1^) for 300 h, and the FTIR spectra were recorded. The FTIR spectra of the blank PVC film are represented in Figure 3. A low concentration of the additives was used to reduce the risk associated from the possible leakage of organotin compounds into soil and ground water.

The intensities of the OH (3500 cm^−1^), C=O (1722 cm^−1^), and C=C (1602 cm^−1^) peaks were monitored during the irradiation of PVC and compared with the corresponding ones before irradiation. The indices (*I*_s_) of these groups were calculated using Equation (1), which gives the ratio of the absorbance of the functional group (*A*_s_) to that of the reference (*A*_r_), (i.e., the C–C bond (1328 cm^−1^) in the PVC chain [37]).

(1) $$I_{s}=A_{s}/A_{r}$$

**Figure 4:**
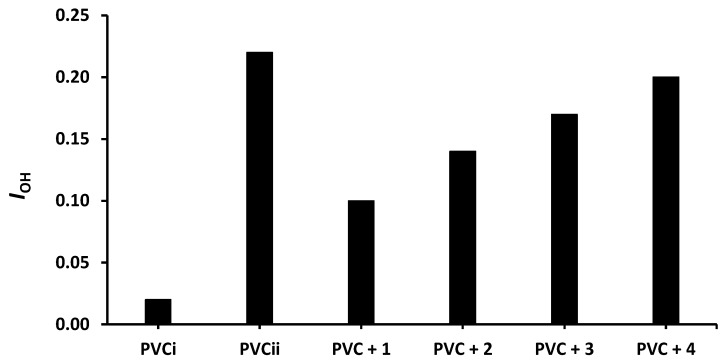
Effect of irradiation (300 h) on the index of the OH group (*I*_OH_) of PVC films containing tin(IV) compounds **1**–**4**. PVC_i_ and PVC_ii_ are the *I*_OH_ of the blank PVC film before and after irradiation, respectively.

**Figure 5:**
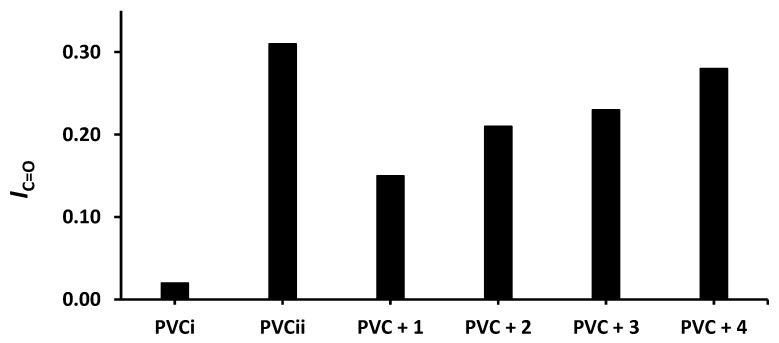
Effect of irradiation (300 h) on the index of the C=O group (*I*_C=O_) of PVC films containing tin(IV) compounds **1**–**4**. PVC_i_ and PVC_ii_ are the *I*_OH_ of the blank PVC film before and after irradiation, respectively.

**Figure 6:**
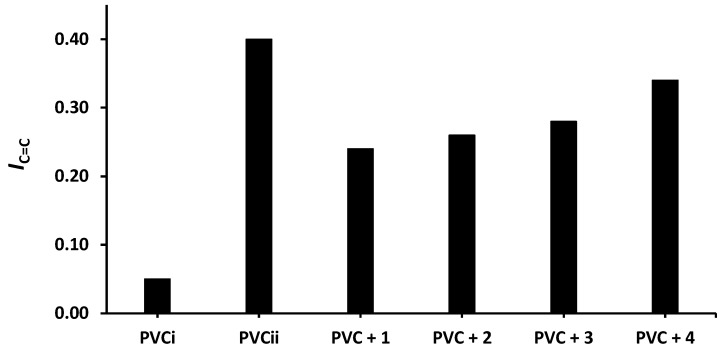
Effect of irradiation (300 h) on the index of the C=C group (*I*_C=C_) of PVC films containing tin(IV) compounds **1**–**4**. PVC_i_ and PVC_ii_ are the *I*_OH_ of the blank PVC film before and after irradiation, respectively.

The changes in *I*_OH_, *I*_C=O_, and *I*_C= C_ after 300 h of irradiation are shown in Figure 4, Figure 5 and Figure 6, respectively. The change in *I*_OH_ is lower for the PVC films containing compounds **1**–**4** compared with that observed for the blank PVC film (Figure 4). For example, the *I*_OH_ of the PVC films is 0.02 before irradiation and increases significantly to 0.22 for the blank PVC film, in contrast to only 0.10 for the PVC + compound **1** film. Similar observations are made for *I*_C = O_ (Figure 5) and *I*_C = C_ (Figure 6). Clearly, compounds **1**–**4** act as PVC photostabilizers. The highly aromatic compound **1** provides the most noticeable photostabilization to the PVC film. Photostabilization of PVC by the Sn(IV) compounds follow the order: **1** > **2** > **3** > **4**.

### 2.3. Weight Loss of PVC

**Figure 7:**
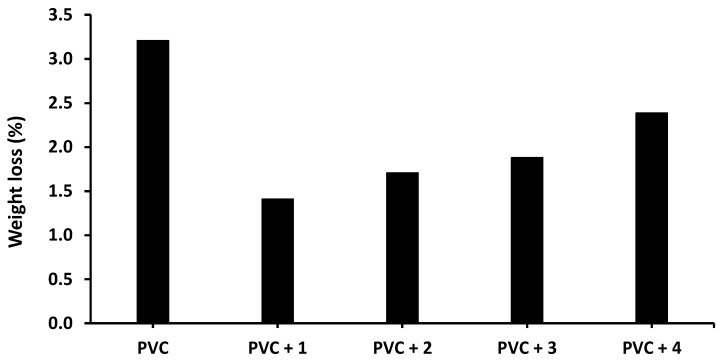
Effect of irradiation (300 h) on the weight loss (%) of PVC films in the absence and presence of tin(IV) compounds **1**–**4**.

The photodegradation of PVC due to irradiation produces volatile and low-molecular-weight by-products [38]. This process causes a weight loss that increases with increasing irradiation time [39]. To check the deterioration of PVC, the films were irradiated for 300 h, and the weight loss due to photodegradation was taken as the difference between the weights of PVC before (*W*_1_) and after (*W*_2_) irradiation, as shown in Equation (2) [40]. The weight loss (%) of PVC in the absence and presence of compounds **1**–**4** is shown in Figure 7.

(2) $$Weight\;loss\;\left(\%\right)\;=\;\left[\left(W_{1}-W_{2}\right)/W_{1}\right]\;\times \;100$$

The weight loss of the blank PVC film is higher than those of the films containing Sn(IV) compounds, and the lowest weight loss is observed for the PVC + compound **1** film. Specifically, the weight loss of the blank PVC film is 3.2% after 300 h of irradiation compared with 1.4% for the PVC + compound **1** film.

### 2.4. Viscosity Average Molecular Weight (${\bar{M}}_{V}$) of PVC

**Figure 8:**
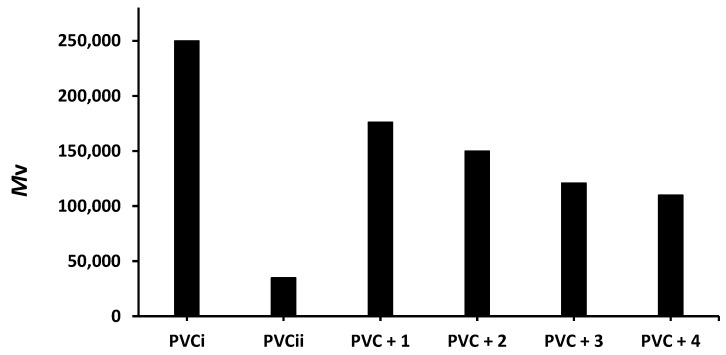
Effect of irradiation (300 h) on the ${\bar{M}}_{V}$ of PVC films containing tin(IV) compounds **1**–**4**. PVC_i_ and PVC_ii_ are the ${\bar{M}}_{V}$ of the blank PVC film before and after irradiation, respectively.

Viscosity measurement of polymeric materials in solution is a versatile method used to determine the ${\bar{M}}_{V}$ of PVC films. The viscosity of a PVC film is expected to decrease after irradiation owing to cross-linking and the branching of polymeric chains [41]. Therefore, the PVC films were dissolved in chloroform after irradiation (300 h), and the viscosity ([η]) was calculated using Equation (3), where *α* and *K* are constants [42]. The decrease in the ${\bar{M}}_{V}$ of PVC after irradiation is shown in Figure 8.

(3) $$\left[\eta \right]=K{\bar{M}}_{V}^{\alpha }$$

The ${\bar{M}}_{V}$ of the blank PVC film is massively reduced from 250,000 to only 35,000 after 300 h of irradiation. On the other hand, the reduction in the ${\bar{M}}_{V}$ of the PVC films containing compounds **1**–**4** is much lower (176,000–110,000). Clearly, the additives reduced PVC photodegradation a significant degree. As in the previous tests, compound **1** leads to the lowest reduction in the ${\bar{M}}_{V}$ of PVC (176,000).

### 2.5. Optical Microscopy of PVC

**Figure 9:**
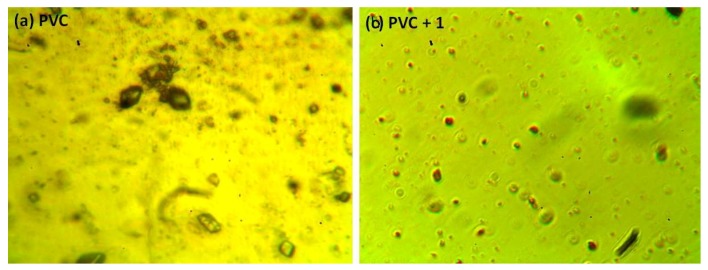
Optical microscopic images of the (**a**) blank PVC film and (**b**) PVC + compound **1** film before irradiation.

**Figure 10:**
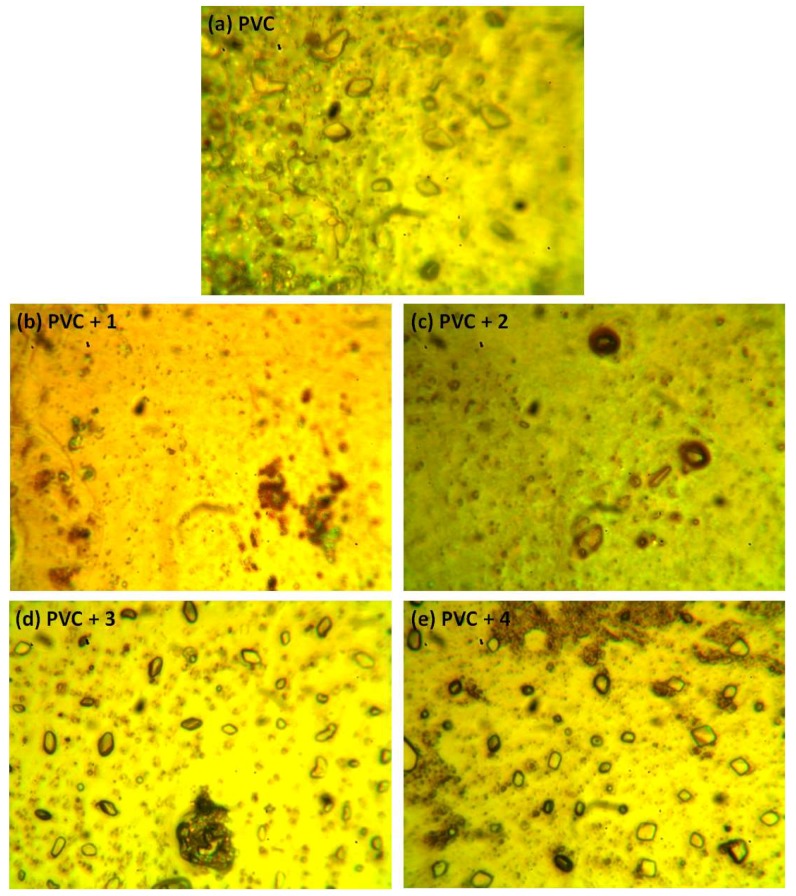
Optical microscopic images of the (**a**) blank PVC film and (**b**–**e**) PVC films containing tin(IV) compounds **1**–**4** after irradiation (300 h).

The photodegradation of PVC leads to surface irregularities, such as cracks, spots, grooves, and discoloration, due to elimination of volatile compounds and chain scission. Such irregularities can be examined using optical microscopy [43,44]. Previous studies proved that the surface of non-irradiated polymeric materials are regular with little or no defects, and are free from cracks and spots [11,15,45]. Figure 9 shows the optical microscopy images for the PVC (blank) and that contains additive **1** before irradiation. The optical microscopic images (400× magnification) of the irradiated blank PVC film and PVC films containing compounds **1**–**4** are shown in Figure 10.

After irradiation, the PVC films show cracks, spots, grooves, and discoloration. However, such defects are fewer in the PVC + compound **1**–**4** films, particularly the PVC + compound **1** film. Organotin compounds **1**–**4** inhibit the evolution of hydrochloride and improve the photostability of the PVC films.

### 2.6. Scanning Electron Microscopy (SEM) of PVC

**Figure 11:**
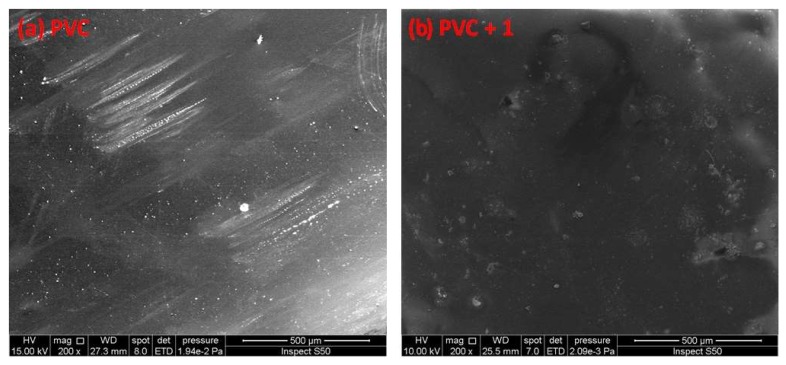
SEM images of the (**a**) blank PVC film and (**b**) PVC + compound **1** film before irradiation.

**Figure 12:**
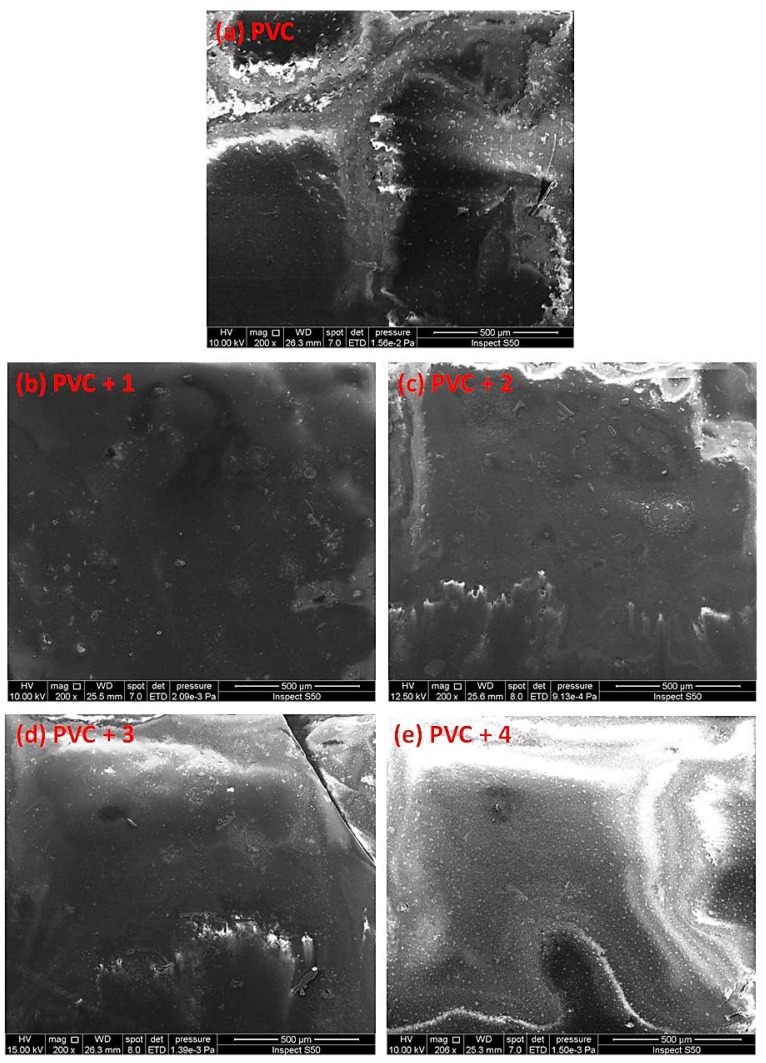
SEM images of the (**a**) blank PVC film and (**b**–**e**) PVC films containing tin(IV) compounds **1**–**4** after irradiation (300 h).

SEM is a useful tool for investigating variation in the surface of polymeric materials. In addition, it can detect the cross-section, homogeneity, shape, and size of particles within material blends [46,47,48,49]. The SEM images of non-irradiated polymeric films were reported to show a high degree of homogeneity, smoothness, and cleanness [50]. Figure 11 shows the SEM images for the PVC (blank) containing additive **1** before irradiation. The PVC films were irradiated for 300 h, and the material surfaces were observed by SEM and are shown in Figure 12.

The damage on the surface of the blank PVC film is more visible than those on the surfaces of the PVC/Sn(IV) blends. The SEM images of the PVC films containing additives, particularly PVC + compound **1**, indicate a less rough surface, smaller cavities, and high degree of homogeneity.

### 2.7. Atomic Force Microscopy (AFM) of PVC

**Figure 13:**
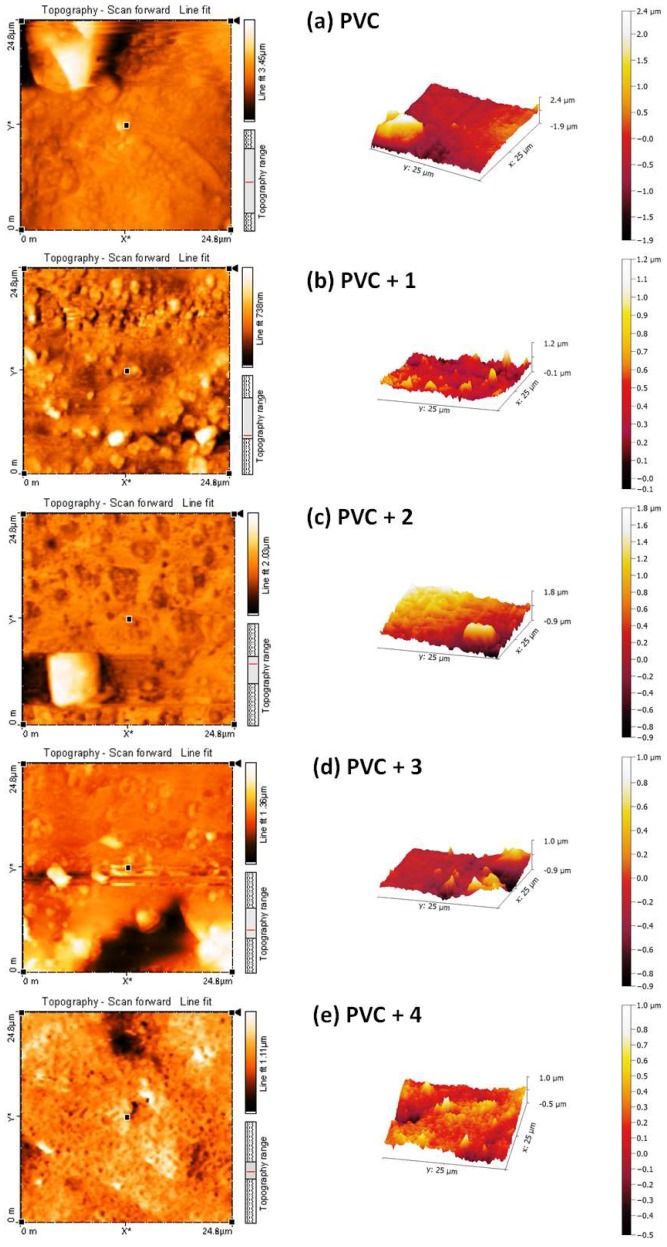
2D and 3D atomic force microscopy (AFM) images of the (**a**) blank PVC film and (**b**–**e**) PVC films containing tin(IV) compounds **1**–**4** after irradiation (300 h).

Atomic force microscopy (AFM) does not involve an electron beam or vacuum, and provides important and useful information about the homogeneity and smoothness of a material’s surface [51]. The AFM images of non-irradiated polymers were reported to show a homogenous and smooth surface [45,50]. To further investigate the surface morphology of the irradiated PVC, AFM images were recorded. The two- and three-dimensional AFM images of the PVC films after irradiation (300 h) are shown in Figure 13. They indicate that the PVC/Sn (IV) blends have more regular, smoother, and less rough surfaces compared with the blank PVC film.

The roughness factor (*R*q) of the blank PVC film is higher (831.4) than those of the PVC/Sn(IV) blends. The *R*q values of the PVC + compound **1**, PVC + compound **2**, PVC + compound **3**, and PVC + compound **4** films are 88.6, 127.8, 226.8, and 383.2, respectively. These lower *R*q values indicate that bond breaking [52] and dehydrochlorination [53] in the PVC/Sn(IV) blends occur at lower rates than in the blank PVC film. Table 1 shows the reduction in *R*q (fold) of PVC for the used additives compared to others that have previously been reported.

### 2.8. Photostabilization Mechanisms of PVC

**Figure 14:**
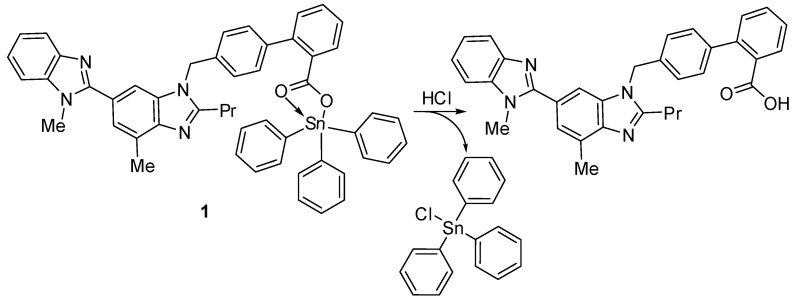
Organotin **1** acts as a hydrogen chloride scavenger.

The synthesized compounds **1**–**4** act as PVC photostabilizers, with the triphenyltin(IV) compound **1** showing the most significant photostabilization effect. They can reduce the photodegradation of PVC through various ways. Sn is acidic and can act as a secondary stabilizer. Therefore, it can capture the hydrogen chloride (i.e., hydrogen chloride scavenger) that evolves during dehydrochlorination (Figure 14). As a result, triphenyltin chloride is produced along with telmisartan (Figure 14). Similar observations with various PVC photostabilizers containing aromatic moieties have been reported [18,33].

**Figure 15:**
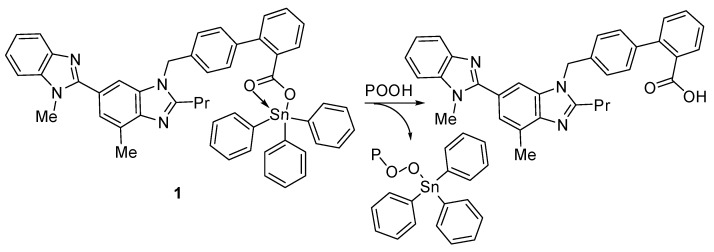
Organotin **1** acts as a peroxide decomposer.

Hydroperoxides are known for their negative impact on PVC chains, which leads to photo-oxidation [54]. The synthesized organotin compounds can decompose hydroperoxides and therefore protect the PVC films against photo-oxidation (Figure 15).

**Figure 16:**
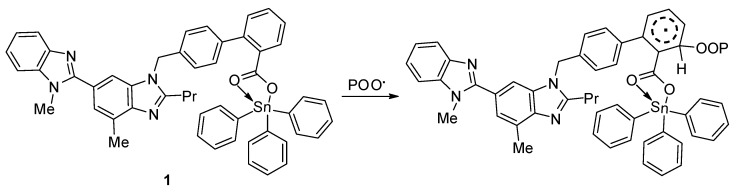
Organotin **1** acts as a radical scavenger.

In addition, peroxide radicals can initiate the photo-oxidation of polymers [55]. Organotin compounds containing aromatic (aryl and heterocyclic) moieties are known radical scavengers. Therefore, they can interact with peroxide radical (chromophore) to produce an excited-state complex [32,33]. Such complexes are highly stable owing to the resonance within the aromatic and heterocyclic moieties (Figure 16).

**Figure 17:**
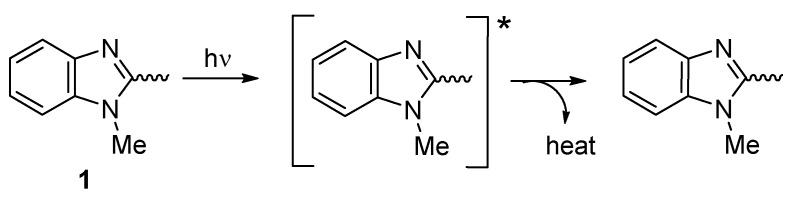
Organotin **1** acts as a UV absorber.

Additives **1**–**4** are aromatic, rich, and can absorb UV light [16]. Their phenyl, aryl, and imidazolyl moieties can absorb UV light, which converts harmful irradiation to harmless energy over time (Figure 17).

**Figure 18:**
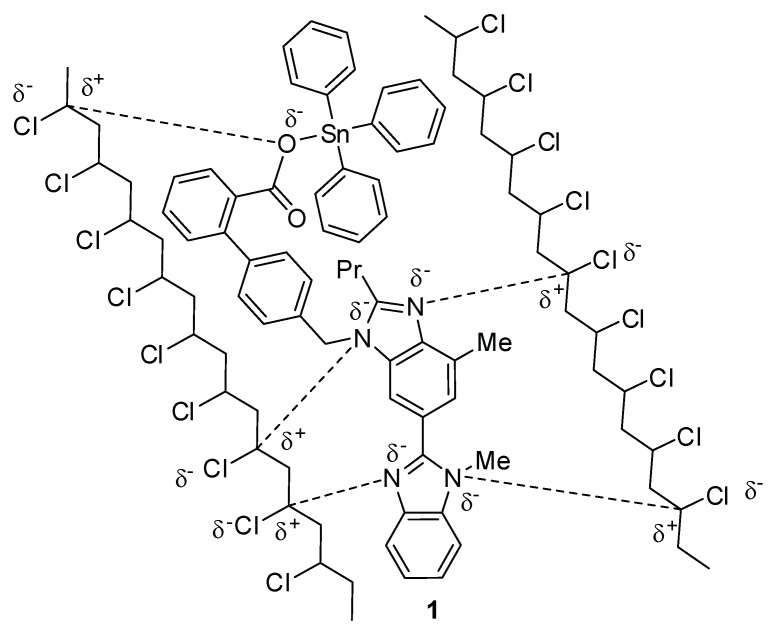
Organotin **1** acts as a primary photostabilizer.

The coordination between the polarized carbons of the C–Cl bonds in PVC and polarized N and O atoms of the imidazole and carboxylate moieties, respectively, of the Sn(IV) compounds can stabilize the polymeric materials (Figure 18). This coordination facilitates the transformation of the excited-state energy to a harmless level in the polymeric chains [16,33].

## 3. Materials and Methods

### 3.1. General

PVC (polymerization degree = 800, *K* value = 67, ${\bar{M}}_{V}$ = *ca*. 250,000) was purchased from Petkim Petrokimya (Istanbul, Turkey). FTIR spectra (400–4000 cm^−1^) were recorded on the Shimadzu FTIR-8300 spectrophotometer (Kyoto, Japan). The PVC surface was inspected using the Meiji Techno microscope (Tokyo, Japan). SEM and AFM images were recorded on a TESCAN MIRA3 field emission SEM system (Kohoutovice, Czech Republic) and Veeco atomic force microscope (Plainview, NY, USA), respectively. The PVC films were irradiated using the QUV-accelerated weathering tester (Q-Panel Company, Homestead, FL, USA) at room temperature. The thickness of the PVC films (40 µm) was adjusted using a Digital Caliper DIN 862 micrometer (Vogel GmbH, Kevelaer, Germany).

### 3.2. Organotin(IV) Compounds ***1**–**4***

Telmisartan organotin(IV) compounds **1**–**4** were synthesized as previously reported [31].

### 3.3. PVC Film Preparation

A solution of PVC (5 g) in tetrahydrofuran (100 mL) was stirred at 25 °C for 30 min; compounds **1**, **2**, **3**, or **4** (0.5 wt%) were added to the PVC solution, and the mixture was stirred at 25 °C for 30 min. The mixture was cast onto glass plates (~40 µm; 4 × 4 cm^2^; 15 holes), and the film was left for 24 h at 25 °C under a vacuum to dry.

## 4. Conclusions

Two telmisartan triorganotin(IV) and two telmisartan diorganotin(IV) compounds were used as photostabilizers to inhibit the photodegradation of PVC upon prolonged UV irradiation of up to 300 h. In the presence of the telmisartan organotin(IV) compounds (0.5 wt%), the rate of photodegradation was much lower. The additives could act as hydrogen chloride and radical scavengers, peroxide decomposers, and primary photostabilizers. The triphenyltin(IV) compound was the most efficient photostabilizer possibly because of its high aromatic content. However, the additives were used at a low concentration, and for the potential commercial use of such additives as PVC photostabilizers, the possibility of leakage of tin and its associated hazards should be tested.

## Figures and Tables

**Table 1:** Reduction in PVC roughness factor (*R*q; fold) in the presence of different additives.

PVC Additive	Reduction in *R*q (fold)	Reference
Telmisartan Sn(IV)	9.4	Current work
Naproxen Sn(IV)	5.2	[30]
Ciprofloxacin Sn(IV)	16.6	[32]
Furosemide Sn(IV)	6.6	[33]
2-(4-Isobutylphenyl)propanoate Sn(IV)	6.2	[22]
Melamine Schiff base	6.0	[11]
Biphenyl-3,3′,4,4′-tetraamine Schiff base	3.6	[13]
1,2,3,4-Triazole-3-thiol Schiff base	3.3	[34]
Polyphosphate	16.7	[29]
